# Which One to Endorse Among the Multitude of Lipid Guidelines?

**DOI:** 10.17925/EE.2015.11.01.32

**Published:** 2015-04-11

**Authors:** Alper Sonmez

**Affiliations:** Associate Professor in Medicine, Gulhane Military Medical Academy, School of Medicine, Department of Endocrinology and Metabolism, Ankara, Turkey

**Keywords:** Dyslipidemia management, atherosclerosis, cardiovascular disease

## Abstract

Dyslipidemia is the major risk factor for atherosclerotic cardiovascular diseases. A multitude of lipid guidelines exist, with several controversies, and the best approach in dyslipidemia management is not clear. The tools and lipoproteins used for risk assessment, whether to use a treatment target and implementing drugs other than statins are all controversial points. Until the time for the publication of an agreeable lipid guideline, physicians should choose their way by considering the advantages and disadvantages of the existing guidelines.

Atherosclerotic cardiovascular diseases (CVD) are the leading cause of death in the West,^1^ and dyslipidemia is the major risk factor for atherosclerosis.^2^ The majority of CVD events would be more susceptible to prevention with effective management for prevention of dyslipidemia. Unfortunately, many patients who have high cardiovascular risk still have unfavourable lipid profiles.^3^ Because the field of lipidology is rapidly growing, medical practitioners need clinical practice guidelines to apply evidence-based medicine daily in dyslipidemia management. However, today, physicians are bewildered by a multitude of guidelines written by different professional societies, which have more diversities than commonalities.^4–11^ This seems to be a critical reason for the suboptimal management of dyslipidemia-related cardiovascular risk.

There are important differences between the current lipid guidelines, such as the recommended lipoprotein measurements for risk assessments, tools used to estimate CVD risk and whether to use a treatment target or to implement drugs other than statins.^12^ Low-density lipoprotein cholesterol (LDL-C) is the primary target in most of the current guidelines.^4–9^ However, the International Atherosclerosis Society (ISA) favours non-high-density (HDL)-C as the primary target of dyslipidemia management.^10^ Moreover, the American Association of Clinical Endocrinologists (AACE) recommends calculation of non-HDL-C in the presence of elevated triglycerides, diabetes, insulin resistance or coronary artery disease.^11^ Indeed, the predictive power of non-HDL-C is better than that of LDL-C in various clinical situations,^13,14^ and it is also superior to LDL-C measurement, being able to be performed in a non-fasting state.^15^ It seems that using non-HDL-C in risk assessment is practical, as recommended by the ISA and AACE guidelines.^10,11^

Another controversial point surrounding the current guidelines is the method of CVD risk assessment. CVD risk is defined as the risk of both mortality and morbidity in the majority of these guidelines. The joint guideline of the European Society of Cardiology (ESC) and the European Atherosclerosis Society (EAS) are more robust than the other guidelines, calculating the fatal CVD risk only.^5^ Also, most current guidelines calculate 10-year risk of CVD. However, ISA recommends measuring the lifetime risk.^10^ Most recently, the American College of Cardiologists (ACC) and American Heart Association (AHA) also recommend measuring 30-year risk of CVD events in patients aged of 20–59.^7^ It is essential to measure long-term or lifetime risk, especially in young adults, who seem to have minimal risk using only 10-year risk. Moreover, these guidelines only reflect the data of the cohort populations enrolled, so the prudent physician must discover whether a tool considers the race, sex and age of an individual patient before using it for risk estimation.

The latest ACC/AHA guidelines further aggravate the ongoing debate by taking a significantly different approach to lipid management in that the authors of have only considered ‘good-quality’ evidence, such as randomised controlled trials, meta-analyses and systematic reviews.^7^ Several common practices of the previous guidelines were not supported, with the evidences behind them being considered not ‘good enough’. The guideline simply defines four specific groups of patients who benefit from moderate- or high-dose statin therapy, recommending no drugs other than statins to treat high LDL-C levels. The new guideline’s most controversial approach is to abandon specific LDL-C targets and recommend a ‘fire-and-forget’ strategy. This approach, also endorsed for chronic kidney disease patients in the recent Kidney Disease: Improving Global Outcomes (KDIGO) guidelines,^8^ has been highly criticised by many other authorities, especially in light of the results of the IMPROVE-IT study.^16^ The other problem with the new ACC/AHA guidelines is that the algorithm used to assess the 10-year risk of the CVD risk significantly overestimates the risk,^17^ indicating start of statin therapy for dyslipidemia patients without atherosclerotic CVD, who are otherwise not candidates of lipid-lowering treatment. Moreover, the guideline does not address persons aged <40 years or aged >75 years, racial and ethnic groups besides whites and African Americans or persons who have conditions such as CKD. Accordingly, it is not possible to say that the new ACC/AHA guideline meets all needs of physicians in its current form.

In conclusion, the existing dispute between the leading authorities backing the current lipid guidelines must be resolved, ideally by organizing a working group in dyslipidemia management and integrating existing guidelines into a general consensus document. However, owing to the number of controversial areas, this is not likely to occur soon. Until it does, physicians working in the field must make their own decisions for each patient by taking into consideration the advantages and disadvantages of the existing guidelines.
